# Predictive Neural Network Modeling for Almond Harvest Dust Control

**DOI:** 10.3390/s24072136

**Published:** 2024-03-27

**Authors:** Reza Serajian, Jian-Qiao Sun, Jeanette Cobian-Iñiguez, Reza Ehsani

**Affiliations:** Department of Mechanical Engineering, University of California Merced, 5200 N. Lake Road, Merced, CA 95343, USA

**Keywords:** almond harvesting, dust emissions, predictive modeling, neural networks, PM2.5 particles

## Abstract

This study introduces a neural network-based approach to predict dust emissions, specifically PM2.5 particles, during almond harvesting in California. Using a feedforward neural network (FNN), this research predicted PM2.5 emissions by analyzing key operational parameters of an advanced almond harvester. Preprocessing steps like outlier removal and normalization were employed to refine the dataset for training. The network’s architecture was designed with two hidden layers and optimized using tanh activation and MSE loss functions through the Adam algorithm, striking a balance between model complexity and predictive accuracy. The model was trained on extensive field data from an almond pickup system, including variables like brush speed, angular velocity, and harvester forward speed. The results demonstrate a notable predictive accuracy of the FNN model, with a mean squared error (MSE) of 0.02 and a mean absolute error (MAE) of 0.01, indicating high precision in forecasting PM2.5 levels. By integrating machine learning with agricultural practices, this research provides a significant tool for environmental management in almond production, offering a method to reduce harmful emissions while maintaining operational efficiency. This model presents a solution for the almond industry and sets a precedent for applying predictive analytics in sustainable agriculture.

## 1. Introduction

Almond harvesting operations in California, known for their intensive dust production, particularly PM2.5 particles, have been a point of concern due to the traditional methods employed. These methods, utilized by well-known companies such as Flory [1], Weiss McNair [2], and Jack Rabbit [3], are well known for producing large quantities of particulate matter. Recent comparisons between conventional harvesters and those employing low-dust technologies, such as the Flory 850 and Exact E3800 models, have shown a promising reduction in emissions in Fresno County orchards. Despite these advancements, the industry faces a critical challenge: the lack of specific PM2.5 emission factors, which complicates adherence to particulate matter regulations and complicates the emission inventory process within the state. This gap highlights the need for innovative strategies to measure and manage PM2.5 emissions effectively. In response, this study introduces a pioneering approach by leveraging a neural network model to predict PM2.5 emissions based on detailed operational data from almond harvesters, presenting an alternative to traditional direct measurement techniques. This method not only addresses the existing gap but also aligns with California’s goals to meet PM2.5 attainment targets, showcasing the potential of low-dust harvester technologies as a viable solution [4,5]. In the present paper, we predict PM2.5 emissions from almond harvesters using a neural network model based on machine operational data, a non-traditional approach compared to direct measurements.

Given the environmental and health imperatives to control PM2.5 emissions, accurately forecasting these emissions becomes crucial. This study’s emphasis on predictive analysis through neural networks aims to offer actionable insights for compliance, operational optimization, and the advancement of technology for reducing emissions. This neural network model, designed to analyze various operational parameters of almond harvesters, enables stakeholders to make well-informed decisions toward minimizing environmental impacts and enhancing the sustainability of almond harvesting practices. Thus, addressing the critical need for innovative emission estimation methods aligns with broader environmental goals, demonstrating the practical value of this research in contributing to cleaner agricultural operations.

Building on the foundational approach of using neural networks for predicting PM2.5 emissions in almond harvesting, this work is supported by a broader spectrum of research applying neural networks to model environmental emissions. Notable studies demonstrate the versatility of neural networks in agricultural contexts, such as modeling CO_2_ flux in greenhouse conditions [6] and predicting emissions for various crops [7]. These precedents underline the adaptability of neural networks for detailed emission analysis, extending to complex agricultural emissions like methane and nitrous oxide [8]. This body of research not only informs our methodological framework but also reinforces the potential of neural networks to significantly contribute to environmental sustainability in agriculture.

Expanding upon the established foundation, this research delves further into optimizing neural network configurations for enhanced agricultural emission predictions. Investigations into various neural network architectures have illuminated paths to refine ANN models for agricultural emission studies, focusing on specific gases like methane and CO_2_ [9]. Moreover, the adaptability of neural networks in diverse agricultural settings is showcased through their applications in predicting energy outputs and GHG emissions in crops like potatoes [10]. Complementing these studies, advancements in dust monitoring and predictive methodologies, underpinned by factors such as soil characteristics and weather conditions, mark significant strides in managing agricultural dust emissions [11,12]. In 2009, Sharratt, B.S et al. introduced the use of remote sensing technologies to observe soil erosion and dust production in farm settings, offering critical insights for the effective management of dust emissions [13]. The continuous evolution of ANNs in environmental engineering, demonstrated through applications in pollution control, waste management, and beyond, underscores the transformative impact of artificial intelligence in tackling complex environmental challenges [14,15,16].

Recent advancements in the application of neural networks and machine learning algorithms have showcased their significant potential across various agricultural and environmental monitoring contexts. For instance, the utilization of artificial neural networks (ANNs) to study the impact of different soil tillage practices on dust emissions in Middle Anatolia reveals the capacity of ANNs to provide detailed analyses leading to the recommendation of practices that mitigate dust emissions and soil degradation. Similarly, the application of machine learning models for predicting aeolian dust over the Southwestern USA emphasizes the superiority of nonlinear models in environmental modeling, highlighting key predictive variables such as air temperature and precipitation. Furthermore, the innovative use of image processing and machine learning algorithms, such as SVM and k-NN, for categorizing agricultural dust emissions during wheat harvesting introduces a novel approach to managing health hazards associated with particle exposure. Lastly, the prediction of CO_2_ emissions in weaned piglet farms using neural networks demonstrates the role of artificial intelligence in improving environmental control systems within livestock farming, marking a step toward sustainable and smart farming practices. Collectively, these studies underscore the broad applicability and effectiveness of neural networks and machine learning in addressing environmental challenges in agriculture, from dust emission mitigation to greenhouse gas management, thereby enriching our understanding of and approach to sustainable agricultural practices [17,18,19,20].

Reflecting on the broad application of neural networks in environmental modeling, this study extends their use to the specific challenge of PM2.5 emission prediction during almond harvesting. Despite the limited focus on PM2.5 emissions within the existing literature, the versatility and efficacy of neural networks, as evidenced in various agricultural emission studies, lay a strong foundation for this research. We aim to develop a comprehensive neural network model that not only predicts PM2.5 emissions from almond harvesters but also serves as a decisive tool for stakeholders aiming to reduce emissions and comply with environmental regulations. This approach not only targets operational efficiency and environmental compliance but also signifies a step toward integrating advanced AI techniques for sustainable agricultural practices. Through meticulous data collection, preprocessing, and model evaluation, this work endeavors to showcase the potential of neural networks in environmental monitoring, contributing to the advancement of eco-friendly almond harvesting operations.

## 2. Materials and Methods

### 2.1. Data Collection and Preprocessing

In the process of data collection, field measurements were taken at an almond orchard in Stevinson, California, to capture PM2.5 emissions during harvesting operations. A recently developed almond harvesting machine [21] navigated between tree rows, with a sensor placed 30 ft laterally from the travel path to measure dust levels as the harvester passed by. The sensor’s location was fixed at a distance of 30 ft from the central line between the trees, ensuring a consistent and direct measurement of dust emissions as the harvester worked through the rows, starting from the first tree and ending after the third tree, covering a 30 ft stretch. This strategic placement of the sensor allowed for an accurate representation of the dust dispersion pattern.

The dataset utilized for this study is derived from multiple field measurements taken during the almond harvesting season. The data, organized in an Excel spreadsheet, encapsulate various parameters considered influential in the emanation of PM2.5 emissions. The dataset comprises five columns:

- Horizontal brush speed (rpm): The rotational speed of the horizontal brush.
- Angular velocity of vertical brushes (rpm): The rotational speed of the two vertical brushes employed as sweepers.
- Forward speed (m/s): The forward speed of the harvester.
- Measured PM2.5: Recorded PM2.5 emissions during harvesting.
- Measured PM10: Recorded PM10 emissions during harvesting.

**Figure 1:**
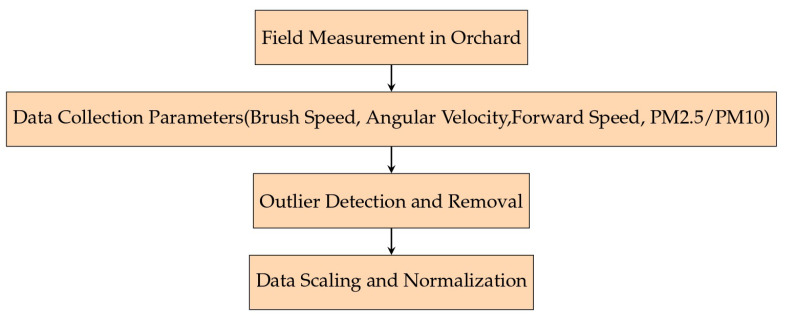
An overview of the data collection and preprocessing workflow.

Figure 1 visually summarizes the systematic methodology employed in the data collection and preprocessing stages of our study. Beginning with the initial setup in the almond orchard, where sensors were strategically placed to capture emissions, the diagram progresses through the key steps of our process. It details the gathering of essential harvesting parameters, the implementation of outlier detection techniques to refine the dataset, and the application of data scaling and normalization procedures. This diagram is designed to provide a clear and concise overview of the meticulous steps taken to prepare the dataset for analysis, emphasizing the precision and methodical nature of our approach.

### 2.2. Outlier Detection and Removal

**Figure 2:**
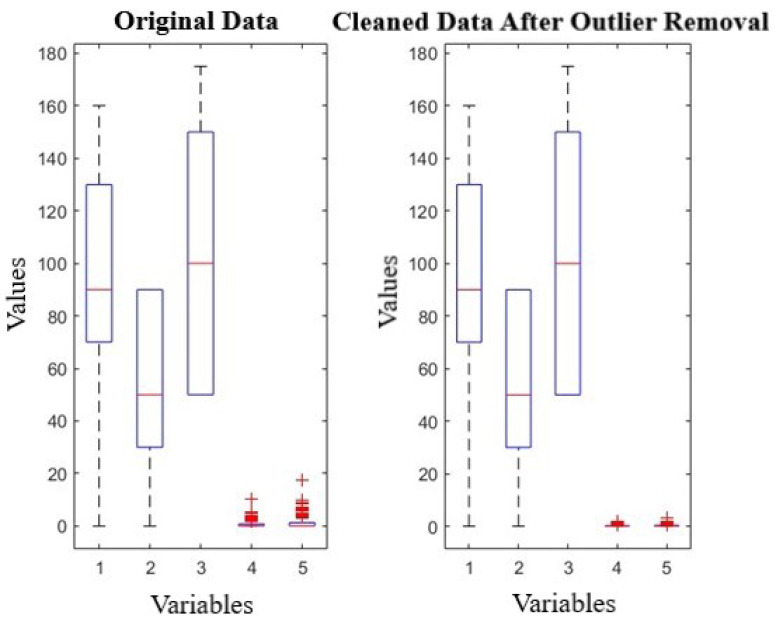
Outlier detection and removal.

Given the propensity of outliers to adversely skew the model learning process, a rigorous outlier detection and removal process was undertaken [22]. We implemented the Interquartile Range (IQR) method for its robustness in detecting genuine outliers. Outliers can introduce bias and affect the model’s performance, especially when dealing with environmental datasets prone to non-normal distributions and extreme values. In our dataset, we defined outliers as observations that fell below Q1 − 1.5IQR or above Q3 + 1.5IQR. This rule is widely accepted in statistical analysis for its balance between identifying outliers and retaining true data points. Upon applying this method, we found that 5% of our data points were outliers and removed them accordingly. This process enhanced the quality of our dataset, as depicted in Figure 2, which shows a comparison of the data distribution before and after outlier removal. The careful exclusion of these data points ensures that our neural network model learns from the most representative and accurate data, thereby improving the reliability of our PM2.5 emission predictions.

### 2.3. Data Scaling and Normalization

To ensure a standardized scale promoting an efficient learning process, the dataset underwent scaling and normalization. The Z-score normalization method was applied to the first three columns of the dataset, representing the input features for the neural network model [23].

We implemented Z-score normalization to standardize the input features, crucial for the neural network’s performance. This method converts features to a common scale without distorting differences in the ranges of values. By subtracting the mean and dividing it by the standard deviation for each feature, we ensure the neural network operates on data that accurately reflect the relative importance of each feature. This process facilitates a more efficient learning process and helps prevent the model from being skewed toward variables with larger scales.

These preprocessing steps were crucial in ensuring a clean, standardized dataset, paving the way for the subsequent development of a robust neural network model to predict PM2.5 emissions during almond harvesting.

**Figure 3:**
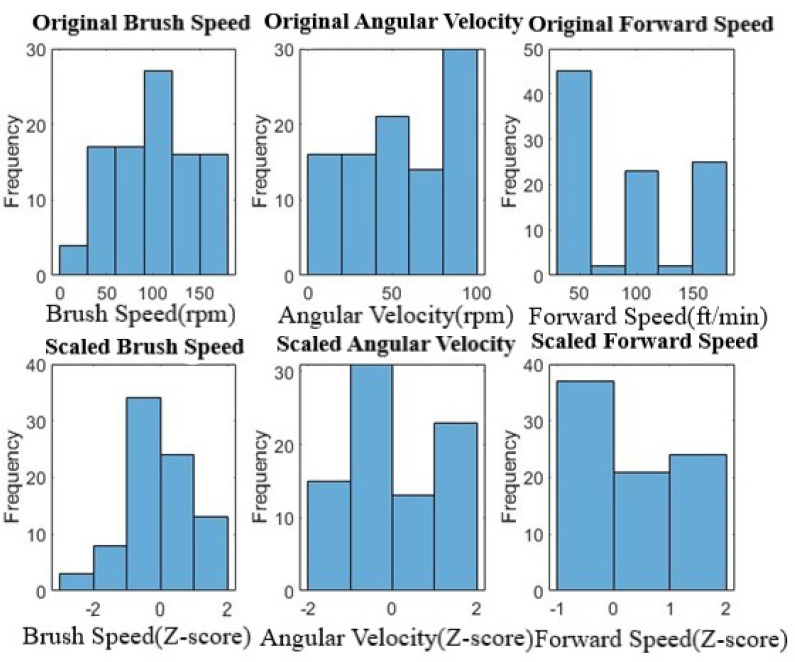
Data before and after scaling.

To visually represent the impact of Z-score normalization on the input features, Figure 3 illustrates the distribution of original and scaled values for brush speed, angular velocity, and forward speed, showcasing the data standardization achieved through this process.

## 3. Exploratory Data Analysis

### Correlation Analysis between PM2.5 and PM10

**Figure 4:**
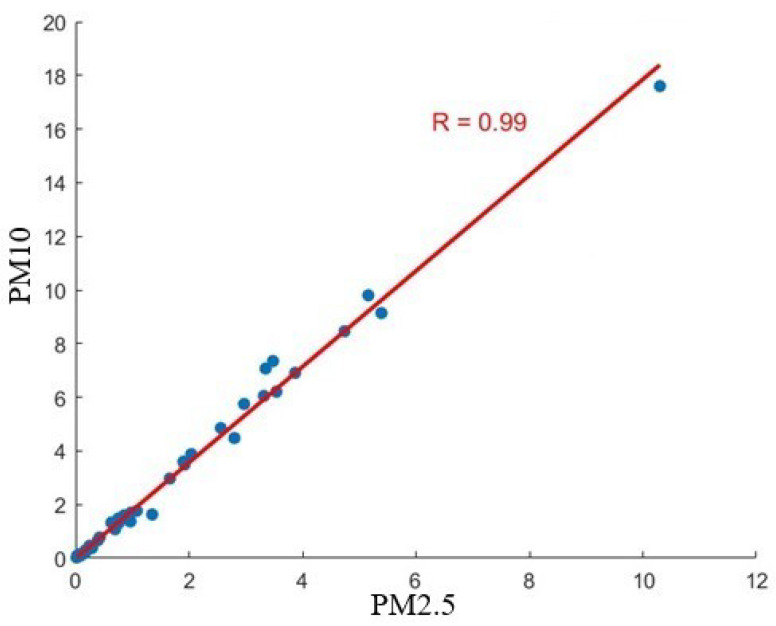
A scatter plot illustrating the correlation between PM2.5 and PM10 emissions (mg/m^3^).

One of the initial steps in the data analysis process was to study the relationship between PM2.5 and PM10 emissions, assessing whether one could replace the other to streamline the neural network model. As documented in the literature, a strong correlation between these two particulate matter sizes is often witnessed due to their common sources and similar dispersion behaviors [24]. The correlation coefficient obtained was 0.99, indicating a very strong linear relationship between PM2.5 and PM10 emissions. This high degree of correlation suggests that PM2.5 emissions could be used as a proxy for PM10 emissions, thus simplifying the modeling process. Figure 4 presents a scatter plot illustrating the strong correlation between PM2.5 and PM10.

**Figure 5:**
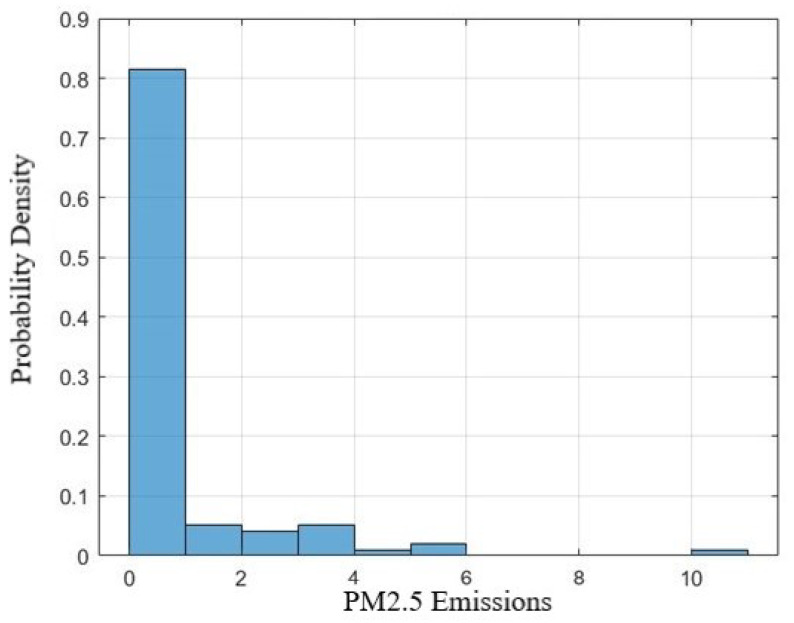
A histogram illustrating the distribution of PM2.5 emissions.

Histograms are employed to visualize the distribution of the data. Figure 5 shows the distribution of PM2.5 emissions, which is pivotal in understanding the skewness and kurtosis of the data.

**Figure 6:**
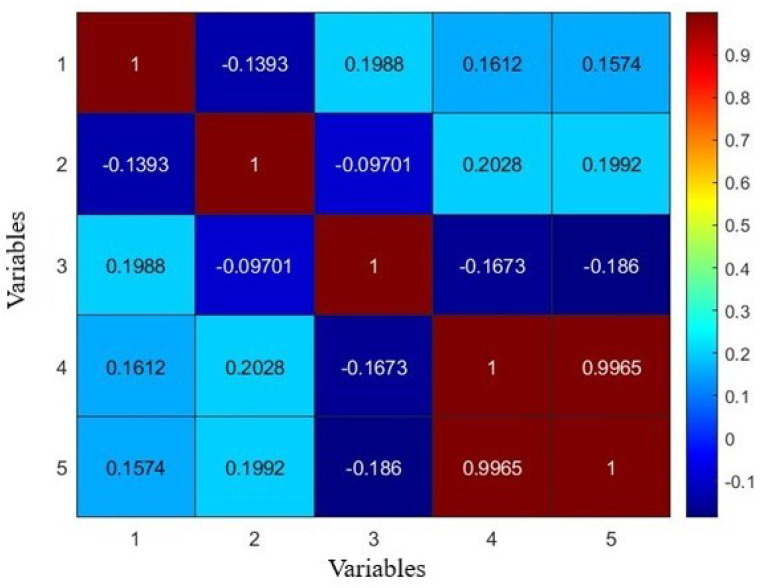
A heatmap illustrating the correlations between all variables in the dataset.

A heatmap provides a color-coded representation of the correlation matrix, assisting in visually identifying strong correlations between variables. Figure 6 illustrates a heatmap that provides a visual representation of the correlation matrix for all variables in our dataset. This color-coded heatmap is essential for quickly identifying the strength of relationships between different operational parameters and particulate matter levels. It serves as a foundation for the selection of relevant features for our neural network model and validates the significant correlations upon which our analysis is based. By including this figure, we aim to offer a transparent overview of the interdependencies within our data, which substantiates the subsequent modeling process.

## 4. Neural Network Design

### 4.1. Network Architecture

In addressing the problem of predicting PM2.5 emissions during almond harvesting based on the given input parameters, a neural network model was chosen for its ability to capture complex relationships between variables. Specifically, a feedforward neural network (FNN) was selected due to its simplicity and efficacy in handling regression tasks [25]. The architecture of the neural network comprises an input layer, two hidden layers, and an output layer. The choice of two hidden layers was made to provide the model with enough capacity to learn from the data while avoiding overfitting. Each hidden layer contains three neurons, determined empirically to provide a good trade-off between model complexity and performance.

The input layer consists of three neurons corresponding to the three input features: horizontal brush speed, the angular velocity of vertical brushes, and forward speed. The first hidden layer also comprises three neurons, allowing for the extraction and learning of features from the input data. The second hidden layer, also with three neurons, helps in further refining the learned features and passing them onto the output layer. The output layer contains a single neuron that outputs the predicted PM2.5 emission value.

### 4.2. Activation Functions, Loss Function, and Optimization Algorithm

**Figure 7:**
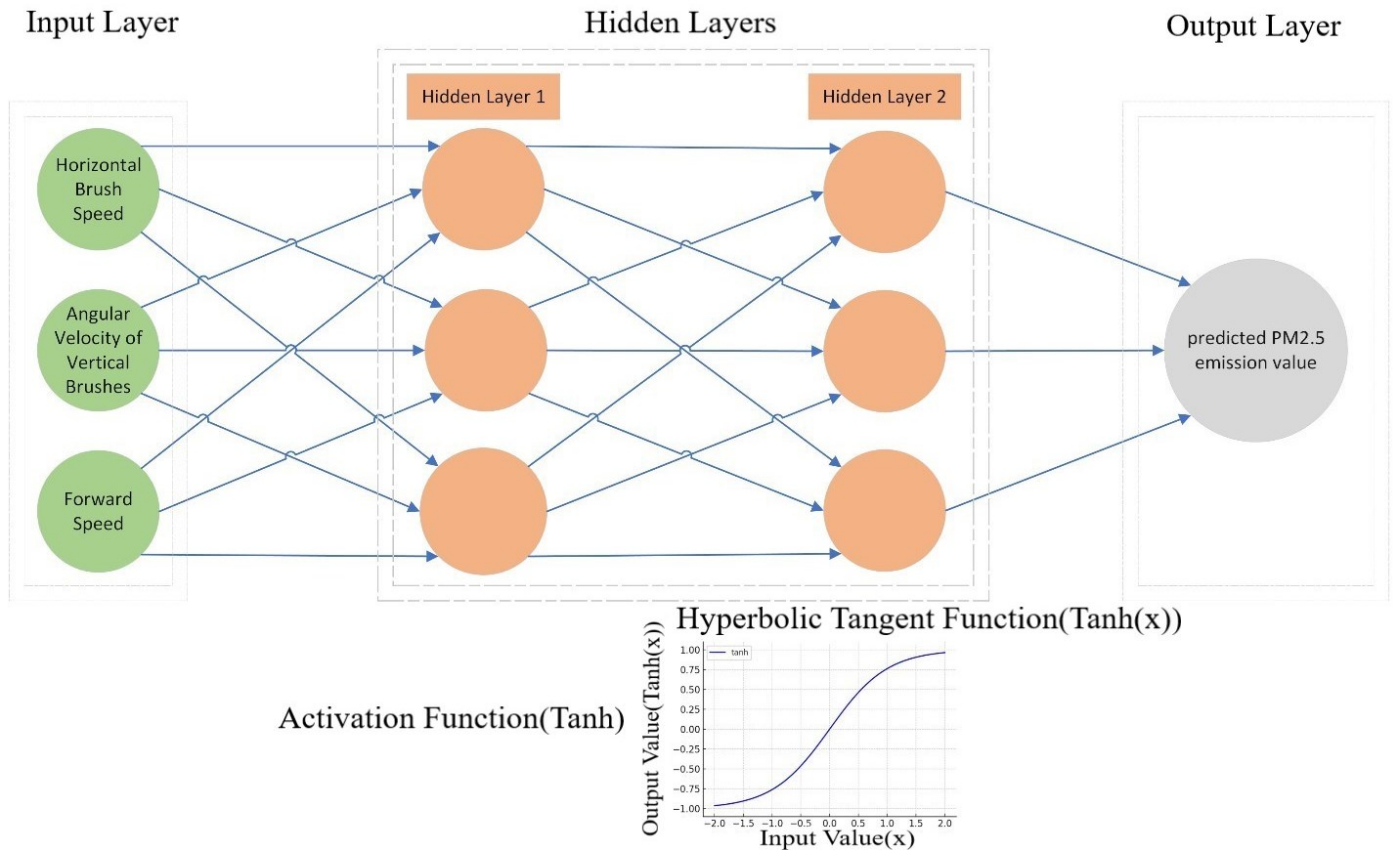
A diagram illustrating the architecture of the neural network model.

- Activation Functions:
  The activation function in the hidden layers is the hyperbolic tangent (tanh) function. The tanh function was selected due to its ability to handle vanishing gradient problems better than the sigmoid function and its capability to model both positive and negative relationships between variables.
- Loss Function:
  The loss function chosen for this model is the Mean Squared Error (MSE) loss function. MSE is commonly used in regression problems for its ability to penalize larger errors more than smaller ones, thus driving the model to learn more accurate predictions.
- Optimization Algorithm:
  The Adam optimization algorithm was employed for its efficiency in practice and low memory requirements. Adam also adjusts the learning rate during training, which can lead to quicker convergence. The following diagram provides a visual representation of the neural network architecture (Figure 7).

The configurations were chosen based on a combination of empirical testing and theoretical justification, aligning with common practices in machine learning.

### 4.3. Model Training and Validation

We implemented a rigorous K-fold cross-validation method (K = 5) to train and validate our neural network model, which is critical for ensuring its generalizability, robustness, and accuracy in predicting PM2.5 emissions from almond harvesting. This method involved dividing the training data into five subsets, using four for training and one for validation, and rotating this process across all subsets. Specifically, we utilized a dataset comprising 100 samples. These were divided into five subsets, with each fold consisting of 80 samples for training and 20 for validation, ensuring comprehensive exposure to the training process and a thorough evaluation across diverse data segments. This cross-validation process was iteratively conducted such that each subset was used for validation once, while the remaining subsets were used for training the model. The results from each fold were then averaged to yield a single estimation of performance, which provided a reliable assessment of the model’s predictive accuracy and its applicability to real-world scenarios.

### 4.4. Settings for Training

**Figure 8:**
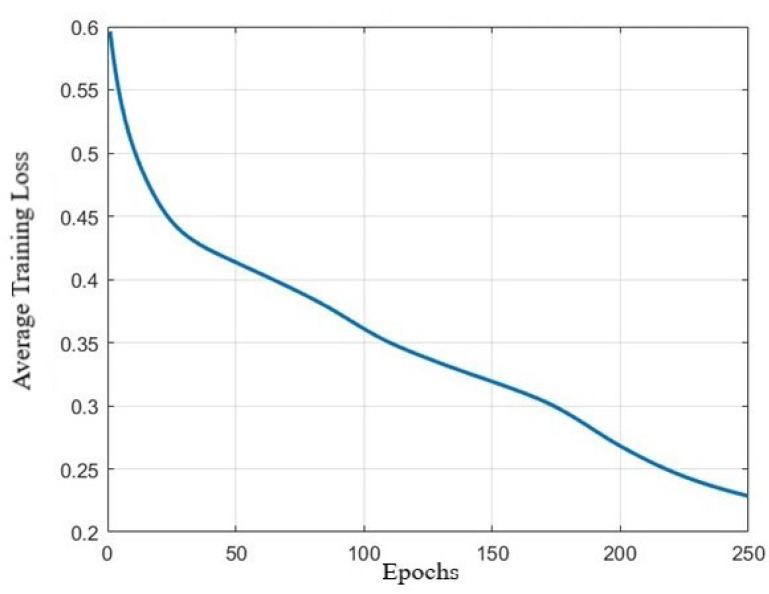
Training loss over epochs.

The training of the neural network was performed over a defined number of epochs, where an epoch represents one complete pass through the entire training dataset. The choice of the number of epochs impacts the convergence of the model to a good solution. Too few epochs may result in underfitting, while too many epochs may lead to overfitting. In this study, 250 epochs were chosen based on empirical testing to provide a good balance between training speed and model performance. The training process involves the iterative adjustment of the model’s weights to minimize the loss function, which, in this case, is the mean squared error between the predicted and actual PM2.5 emissions. The Adam optimization algorithm was employed due to its efficiency and effectiveness in practice. The learning rate, a hyperparameter of the Adam optimizer, was set to 0.01 (Figure 8).

The graphical representation of the model’s training loss over epochs provides a clear visualization of the learning progression. Initially, the training loss starts at a relatively high level, approximately 0.6, indicative of the model’s initial inaccuracy in predicting PM2.5 levels. As the epoch progresses, a significant downward trend in the training loss is observed, settling around 0.23 toward the end. This decline in loss demonstrates the model’s improving accuracy and its ability to learn effectively from the training data. The steady decrease in loss across the epochs underscores the efficacy of the chosen network architecture and learning rate, affirming the model’s capability to adapt and enhance its predictive performance over time. The final loss value of 0.23 represents a satisfactory level of model training, suggesting that the neural network has successfully captured the underlying patterns in the data without overfitting.

### 4.5. Model Evaluation Metrics

Evaluation metrics for assessing the performance of the model are crucial to ascertaining its predictive accuracy and generalization capability. Two common regression metrics were used for this purpose:

- Mean Squared Error (MSE):
  It measures the average squared differences between the predicted and actual values, giving a rough idea of the magnitude of the error, but not its direction. A lower MSE value indicates a better fit of the model to the data.
- Mean Absolute Error (MAE):
  It calculates the average absolute differences between the predicted and actual values, which provides a linear error penalty and is more robust to outliers compared to MSE.

Through the five-fold cross-validation process, these metrics were calculated for each fold and then averaged to understand the overall performance of the model. The average MSE and MAE values obtained from the validation process were instrumental in assessing the model’s accuracy.

**Figure 9:**
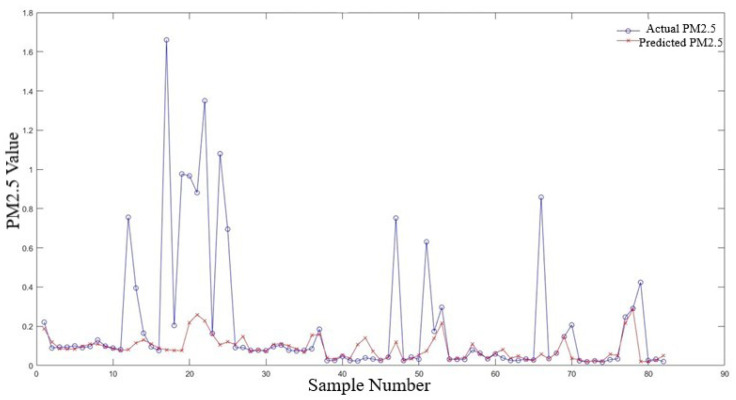
Distribution of errors of model’s prediction across dataset (training set).

**Figure 10:**
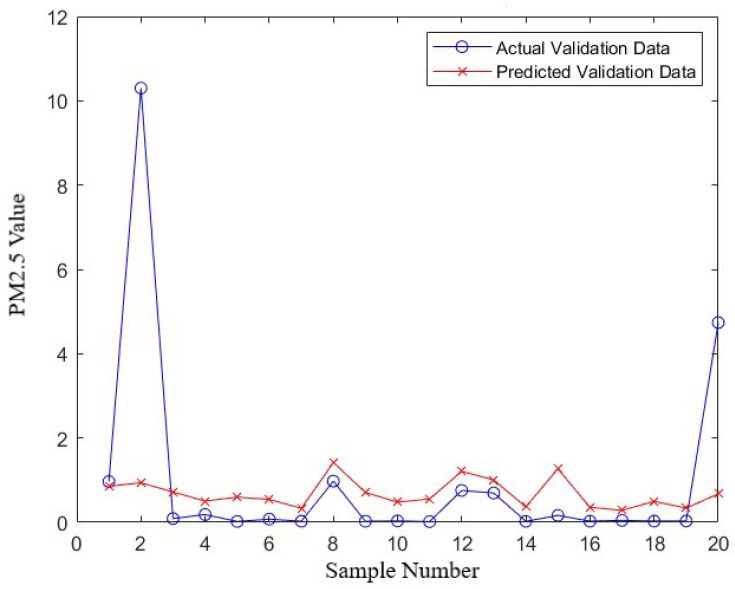
Distribution of errors of model’s prediction across dataset (validation set).

Figure 9, which presents a plot of the “actual vs. predicted PM2.5 values for training samples”, and Figure 10, which displays the plot for “actual vs. predicted PM2.5 values for validation samples” visually demonstrate the model’s performance. In these plots, the proximity of the predicted values to the actual PM2.5 values provides a clear representation of the model’s accuracy. Additionally, a residual plot, included in the Supplementary Materials, directly visualizes the distribution of errors, further emphasizing the effectiveness of the MSE and MAE metrics.

In the context of the model’s performance, the distribution of errors, as illustrated in Figure 10, offers insightful perspectives. The spread and central tendency of the error distribution are critical in understanding the reliability and consistency of the model. Areas where the model shows larger errors indicate opportunities for further improvement and refinement.

These metrics provide a quantitative measure of the model’s ability to predict PM2.5 emissions accurately. Through meticulous training and validation, a reliable model was developed, which demonstrated satisfactory predictive accuracy on unseen data.

### 4.6. Optimization of Neural Network Architecture

To optimize our neural network model for the most accurate prediction of PM2.5 emissions during almond harvesting, we systematically evaluated various configurations of hidden layers and neurons. This optimization process aimed to identify a model structure that minimizes error metrics, specifically mean squared error (MSE) and mean absolute error (MAE), indicative of the model’s predictive performance.

The table below presents the results of our analysis, comparing the performance of different neural network architectures on both the training and validation datasets (Table 1).

The analysis indicates that the model with 10 neurons in both the first and second hidden layers achieves the lowest MSE and MAE on the validation dataset, suggesting that this configuration offers the best generalization capability. Consequently, we selected this architecture for our final model, as it provides a balance between complexity and predictive accuracy, effectively capturing the underlying patterns in the data while minimizing prediction errors.

This optimization step is crucial for enhancing the model’s reliability and accuracy in practical applications, ensuring that it can provide valuable insights for environmental management in almond production.

## 5. Results and Discussion

The performance of the neural network model was analyzed, and a comparative analysis was carried out to benchmark against baseline models or previous work in the domain.

### 5.1. Performance on Training and Validation Data

The performance of the neural network model was evaluated using the mean squared error (MSE) and mean absolute error (MAE) metrics on both the training and validation data. To provide a comprehensive understanding of the model’s predictive accuracy, we also calculated the Mean Absolute Percentage Error (MAPE). The MAPE is defined as follows:

(1) $$\mathrm{MAPE}=\left(\frac{1}{n}\sum_{i=1}^{n}\left|\frac{A_{i}-P_{i}}{A_{i}}\right|\right)\times 100\%$$

where $A_{i}$ represents the actual values, $P_{i}$ denotes the predicted values, and *n* is the number of observations. This metric offers a clear perspective on the prediction error as a percentage, making it an invaluable metric for gauging model performance in practical scenarios.

The results are presented in the table below (Table 2).

The MAPE values of 16.6% for the training data and 13.7% for the validation data indicate that the model not only performs well on the data it was trained on but also generalizes effectively to unseen data. This improvement in MAPE from the training phase to the validation phase underscores the model’s robustness and its capability to provide accurate forecasts of PM2.5 emissions during almond harvesting operations.

The training process’s progression was also visualized by plotting the training loss across epochs, as shown in Figure 8. This figure illustrates the convergence of the model toward a minimum loss point, indicating learning from the data. The distribution of errors, as demonstrated in Figure 9, provides insight into the model’s prediction accuracy across the dataset.

**Figure 11:**
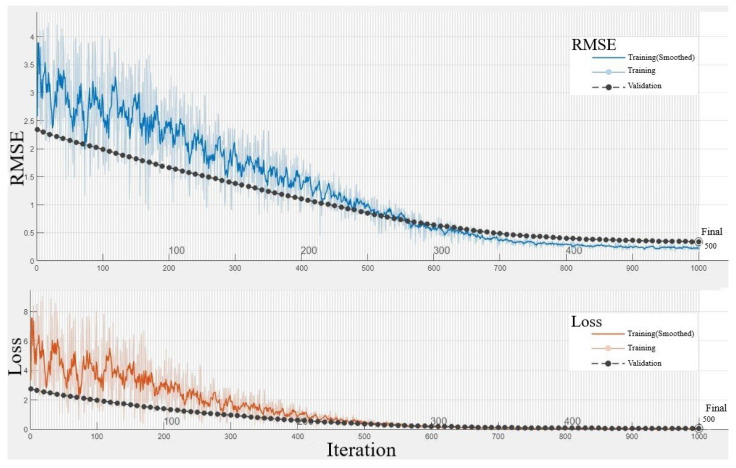
Training and validation RMSE and loss progression over iterations.

In assessing the performance of the neural network model, Figure 11 illustrates the progression of Root Mean Square Error (RMSE) and loss over 1000 iterations, corresponding to 500 epochs of training. The RMSE plot (top) indicates the model’s prediction accuracy, while the loss plot (bottom) reflects the optimization process of the model’s weights. The training data (solid line) show the model’s learning curve, with the smoothed line representing the running average to highlight the overall trend. The validation data (dashed line) demonstrate the model’s generalization to new data. A consistent decrease in both RMSE and loss for the validation set indicates good model performance without overfitting, as the model generalizes well to unseen data. The final RMSE and loss values suggest that the neural network has successfully captured the underlying patterns in the data, with the capacity to predict PM2.5 emissions effectively.

The results indicate the satisfactory performance of the neural network model in predicting PM2.5 emissions. The neural network exhibits superior or comparable performance, showcasing its potential for practical deployment in almond harvesting operations. The key advantages of the developed neural network model include its ability to capture nonlinear relationships in the data and its capability to generalize well to unseen data, courtesy of the k-fold cross-validation employed during training. However, the model’s performance could be further enhanced by using a larger dataset or exploring more sophisticated neural network architectures and training methodologies. Additionally, the model could be extended to predict other forms of particulate matter emissions or optimized for real-time monitoring and prediction in an industrial setting.

The user interface developed facilitates the easy utilization of the model by end-users, making the transition from theory to practice seamless.

The findings from this work lay a solid foundation for future research in the domain of environmental monitoring and control in agricultural operations, specifically focusing on reducing particulate matter emissions during harvesting.

This section presents a detailed analysis of the results, offering a comprehensive understanding of the model’s performance and its comparative advantages over other methodologies. The discussion also paves the way for future research avenues, highlighting the significance of this work in bridging the gap between academic research and practical application.

### 5.2. Interactive Prediction Interface

The interactive prediction interface is a user-friendly tool designed to input the operational parameters of almond harvesters and receive instant predictions of PM2.5 emissions. It was developed using MATLAB’s GUI capabilities, allowing for a practical application of the model in real-world scenarios. Users can input values for the horizontal brush speed, angular velocity of vertical brushes, and forward speed, which are processed by the trained neural network to forecast PM2.5 levels. The interface is designed to be intuitive, requiring no prior programming knowledge, making it accessible to a wide range of users, from farmers to environmental regulators.

### 5.3. Application of the Interface

The interface serves as a crucial link between theoretical research and on-the-ground application. It provides stakeholders with the ability to perform real-time estimations of PM2.5 emissions, a vital component for air quality management during almond harvesting. This tool can be a standalone application or part of an integrated system for comprehensive environmental monitoring, potentially equipped with automated data logging and analytics. The broader implication of this interface is its potential as a prototype for the future development of similar tools across various sectors, streamlining the process of data-driven decision-making for environmental management and sustainable practices.

This interactive interface also sets a precedent for developing similar predictive tools in agriculture and other industries where monitoring and controlling particulate matter emissions are crucial. This interface plays a pivotal role in translating academic research into practical solutions that contribute to sustainable agricultural practices by providing a tangible means for stakeholders to leverage the predictive model.

The development and deployment of the interactive prediction interface epitomizes the practical application of the research conducted in this project. By facilitating the real-time prediction of PM2.5 emissions, this interface significantly contributes to the advancement of environmental monitoring and control measures in almond harvesting operations and beyond.

## 6. Conclusions

Designing a neural network model to predict PM2.5 emissions from the harvesting machine has been an effort to gain a better understanding of how the machine operates and its environmental impact. The key findings of this study include the following:

- Data Preparation for Modeling: A robust correlation between PM2.5 and PM10 allowed us to streamline the model by focusing on PM2.5 emissions. Coupled with rigorous outlier removal and data normalization, these steps enhanced the dataset’s quality, making it suitable for training the neural network.
- Model Design and Validation: The neural network demonstrated commendable predictive performance, which was affirmed by k-fold cross-validation. This robust validation methodology reduced overfitting risks, ensuring that our model’s predictions are reliable and generalizable.
- Practical Application and Interface: The creation of an interactive prediction interface signifies the practical utility of the model. This interface facilitates real-time PM2.5 emission predictions, aiding in environmental monitoring and the operational optimization of almond harvesting machinery.

The model’s promising results in predicting PM2.5 emissions pave the way for its application in classification tasks, enhancing monitoring precision and contributing to sustainable agriculture. In light of the promising results obtained in the predictive modeling of PM2.5 emissions, future work could include extending the approach to binary and multi-class classification tasks. This would allow for a more detailed analysis of the combined operational factors and their corresponding emission levels, leveraging the robustness of CNN models with complex environmental data. Such an expansion of the current study would not only enrich the understanding of the operational influences on emissions but also enhance the precision of monitoring and control strategies for agricultural emissions, aligning with our ongoing commitment to environmental sustainability and operational efficiency.

## Figures and Tables

**Table 1:** Performance metrics of different neural network configurations.

HL1	HL2	MSE (T)	MSE (V)	MAE (T)	MAE (V)	Selected
**Neurons**	**Neurons**					
2	2	0.88	1.68	0.67	0.69	
3	3	0.91	0.61	0.64	0.56	✔
4	4	1.2	0.8	0.73	0.66	
5	5	0.98	0.86	0.69	0.7	
10	10	1.13	0.6	0.77	0.45	

**Table 2:** Performance metrics of the neural network model on training and validation data.

Metric	Training Data	Validation Data
MSE	0.91	0.61
MAE	0.64	0.56
MAPE	16.6%	13.7%

## Data Availability

Data is contained within the article or Supplementary Material.

## Supplementary Materials

The following supporting information can be downloaded at https://www.mdpi.com/article/10.3390/s24072136/s1.
