# Supplementary material for: Predictive Neural Network Modeling for Almond Harvest Dust Control
Source: Sensors (Basel). 2024 Mar 27;24(7):2136. doi: 10.3390/s24072136 (PMC11014124; doi:10.3390/s24072136)
Supplement: Supplementary file 1 [file sensors-24-02136-s001.zip › Paper_supplemet.pdf]

Supplemental Visuals for Understanding PM2.5 Emission Levels

Reza Serajian, Jian-Qiao Sun, Jeanette Cobian-Iñiguez, and Reza Ehsani
Department of Mechanical Engineering, University of California Merced

Introduction

This supplement provides visual representations of different PM2.5 emission levels during almond harvesting operations. These images are intended to enhance the interpretability of the predictive model's outcomes, facilitating an intuitive understanding of the environmental impacts associated with various operational parameters.

High PM2.5 Emissions

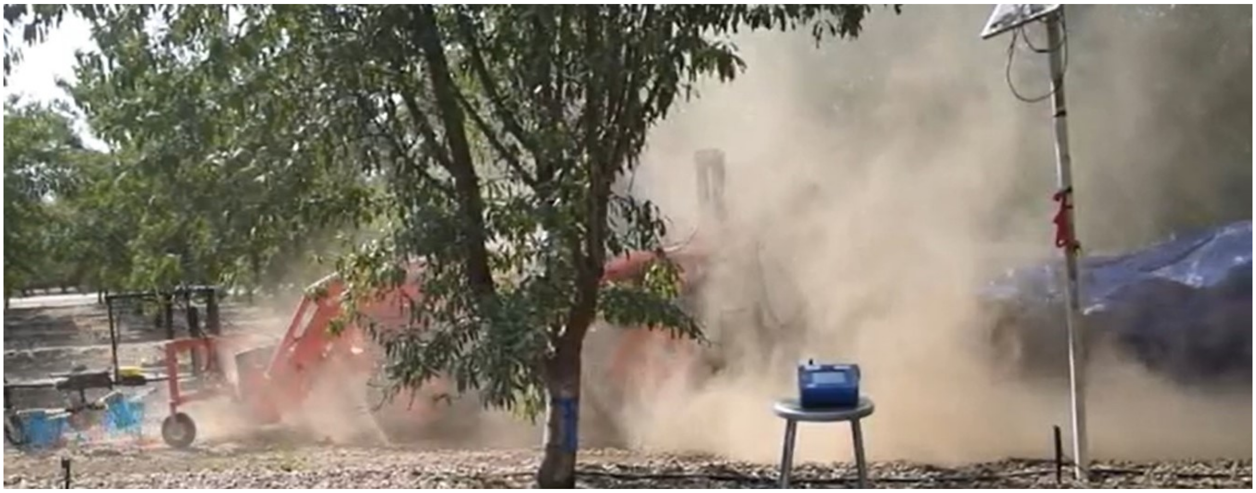

Figure S1: High PM2.5 emissions during peak operational activity with maximum horizontal brush speed, indicating a significant environmental impact.

Medium PM2.5 Emissions

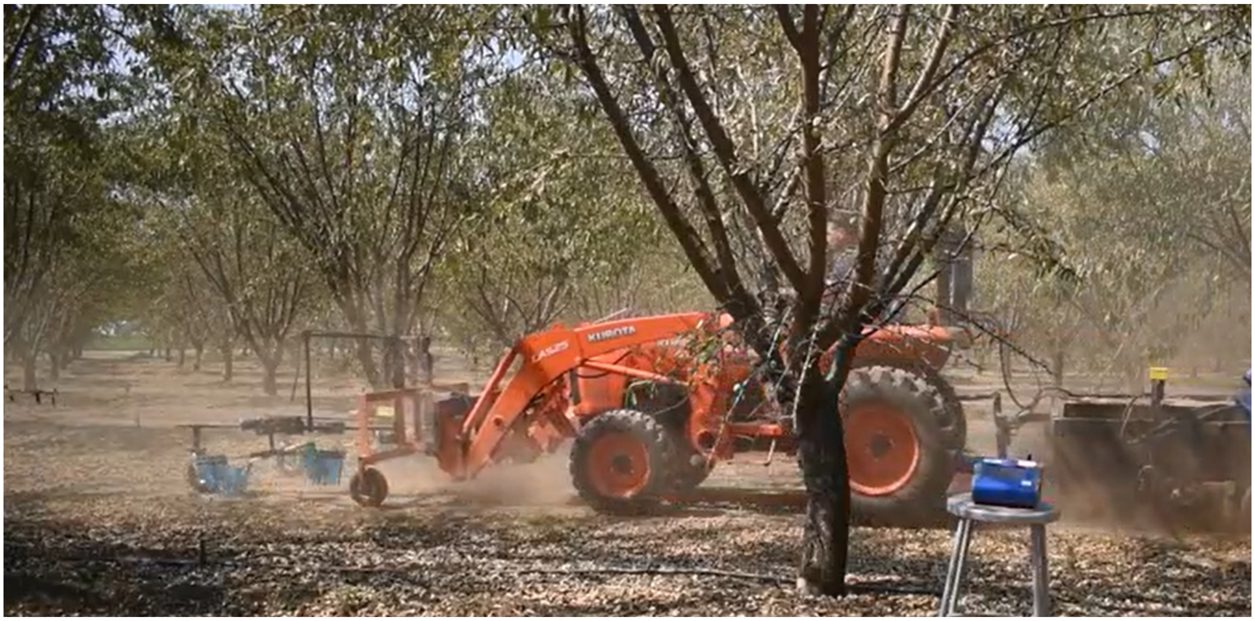

Figure S2: Moderate PM2.5 emissions with adjusted forward speed and vertical brush velocity, showcasing a balanced operational approach.

Low PM2.5 Emissions

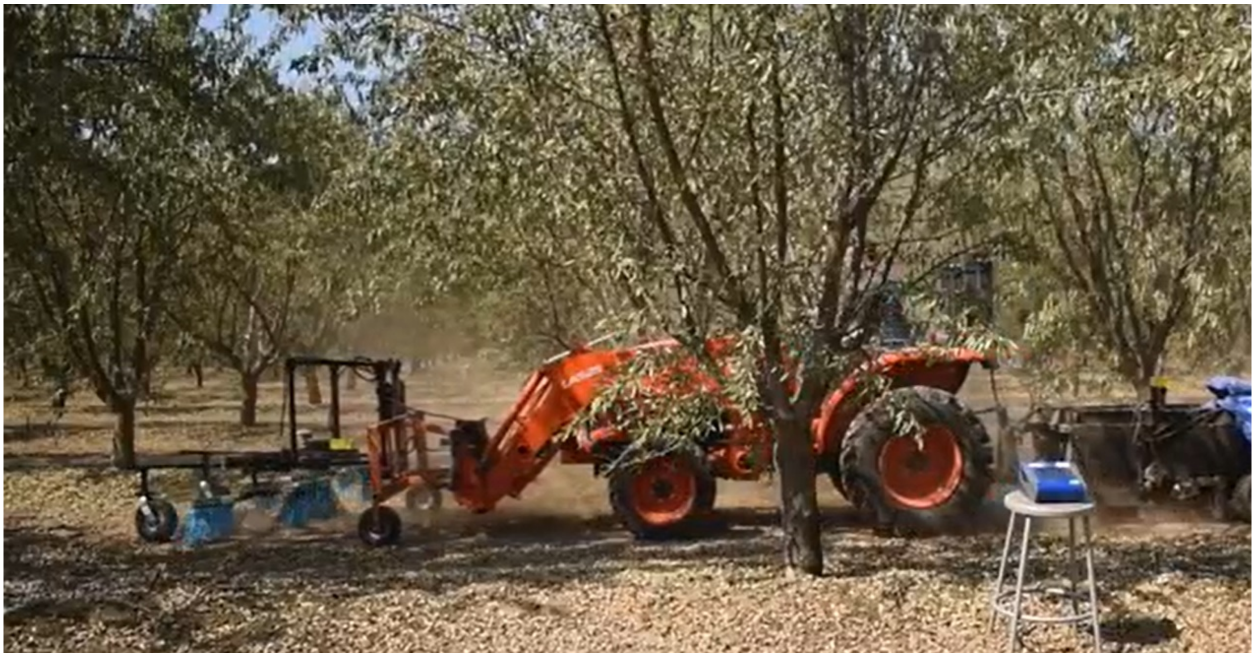

Figure S3: Low PM2.5 emissions reflecting optimized operational parameters for minimal environmental footprint.

Conclusion

These images depict the varying levels of PM2.5 emissions that can be expected under different operational settings of almond harvesting machinery. They serve to bridge the gap between the numerical predictions of our interactive interface and the real-world conditions farmers and environmental regulators might encounter.
